# From monitoring to intervention: a closed-loop digital health framework for gaming disorder

**DOI:** 10.3389/fpubh.2026.1912606

**Published:** 2026-09-04

**Authors:** Longji Li, Lifeng Zhang

**Affiliations:** Strength and Conditioning Center, School of Physical Education, Chengdu Sport University, Chengdu, China

**Keywords:** digital phenotyping, digital therapy, gaming disorder, precision intervention, risk prediction

## Abstract

Gaming disorder has become a significant public health concern and represents a complex, dynamic condition that requires continuous risk monitoring throughout its progression. However, conventional assessment methods, which rely primarily on questionnaires and clinical interviews, are inherently static and retrospective, limiting dynamic monitoring and early risk identification. Although digital phenotyping, artificial intelligence (AI), and digital therapeutics have emerged as promising approaches, existing reviews have largely examined these technologies separately, lacking an integrated framework for intelligent gaming disorder management. This review systematically synthesizes current evidence on digital phenotyping, AI-driven risk prediction, and digital intervention strategies, and integrates them into a “Monitor–Predict–Intervene” closed-loop digital health framework. This study indicates that digital phenotyping enables continuous multimodal monitoring of behavioral, physiological, psychological, and social signals; AI facilitates dynamic risk stratification and trajectory prediction through multimodal data integration; and digital therapeutics, particularly Just-in-Time Adaptive Interventions (JITAIs), deliver personalized interventions based on evolving risk states. Collectively, these technologies establish a closed-loop digital health paradigm that supports continuous risk management across the progression of gaming disorder. Overall, this framework advances gaming disorder management from static assessment to continuous monitoring, from reactive treatment to proactive prevention, and from standardized intervention to individualized precision care, while providing a conceptual foundation and translational roadmap for future digital mental health research and clinical practice. Future research should further address challenges related to data privacy, algorithm interpretability, and clinical implementation to facilitate the development of scalable and intelligent digital health systems.

## 1 Introduction

With the rapid development of digital technology and the continued growth of the gaming industry, video games have become one of the most popular forms of entertainment worldwide (1). However, although gaming provides recreational, educational, and social benefits for most individuals, gaming behavior may gradually develop into a maladaptive pattern characterized by impaired self-control, excessive gaming, and impairment in academic, occupational, and social functioning among vulnerable individuals, ultimately resulting in gaming disorder (2). The World Health Organization (WHO) has officially included gaming disorder in the 11th edition of the International Classification of Diseases (ICD-11), recognizing it as a behavioral addiction (3). There are over 3 billion gamers worldwide, with a prevalence rate as high as 8 to 11% among adolescents and young adults (4, 5). Given its increasing prevalence and its substantial impact on psychological well-being, social functioning, and quality of life, gaming disorder has emerged as a major public health challenge and an increasingly important concern for digital mental health (6).

The identification and assessment of gaming disorder primarily rely on traditional methods such as self-assessment scales, clinical interviews, and cross-sectional surveys (7). While these tools play an essential role in clinical screening and diagnosis, traditional assessment approaches are inherently limited by the static, retrospective, and intermittent nature. Consequently, traditional assessment methods are susceptible to recall bias, limited assessment frequency, and insufficient sensitivity to temporal fluctuations, making it difficult to capture the dynamic evolution of an individual’s risk status over time. The occurrence and progression of gaming disorder are not static but dynamically shaped by the continuous interaction of multiple behavioral, psychological, physiological, and environmental factors, including emotional state, stress levels, sleep quality, social environment, and gaming context (8). Therefore, achieving dynamic and multidimensional monitoring of gaming disorder risk has become an important direction in current research, as it addresses the need for early identification of at-risk individuals while facilitating subsequent risk prediction and personalized intervention.

The rise of digital psychiatry and precision mental health has provided new technological pathways for the risk management of gaming disorder (9, 10). Digital phenotyping can continuously collect behavioral, psychological, physiological, and environmental information through digital terminals such as smartphones, wearable devices, gaming platforms, and social media, thereby enabling real-time monitoring of an individual’s state (11). The development of artificial intelligence (AI) and machine learning technologies has further enhanced the ability to integrate multimodal data and predict risks, making it possible to build early warning systems based on real-world data (12). The core characteristics of gaming disorder are mainly manifested in objectively measurable digital behavioral patterns, such as game duration, login frequency, device usage habits, social interaction, and sleep rhythms (11). Collectively, these emerging digital health technologies and analytical approaches have created unprecedented opportunities for the digital monitoring and management of gaming disorder, supporting the transition toward more objective, data-driven, and personalized mental health care.

**Figure 1:**
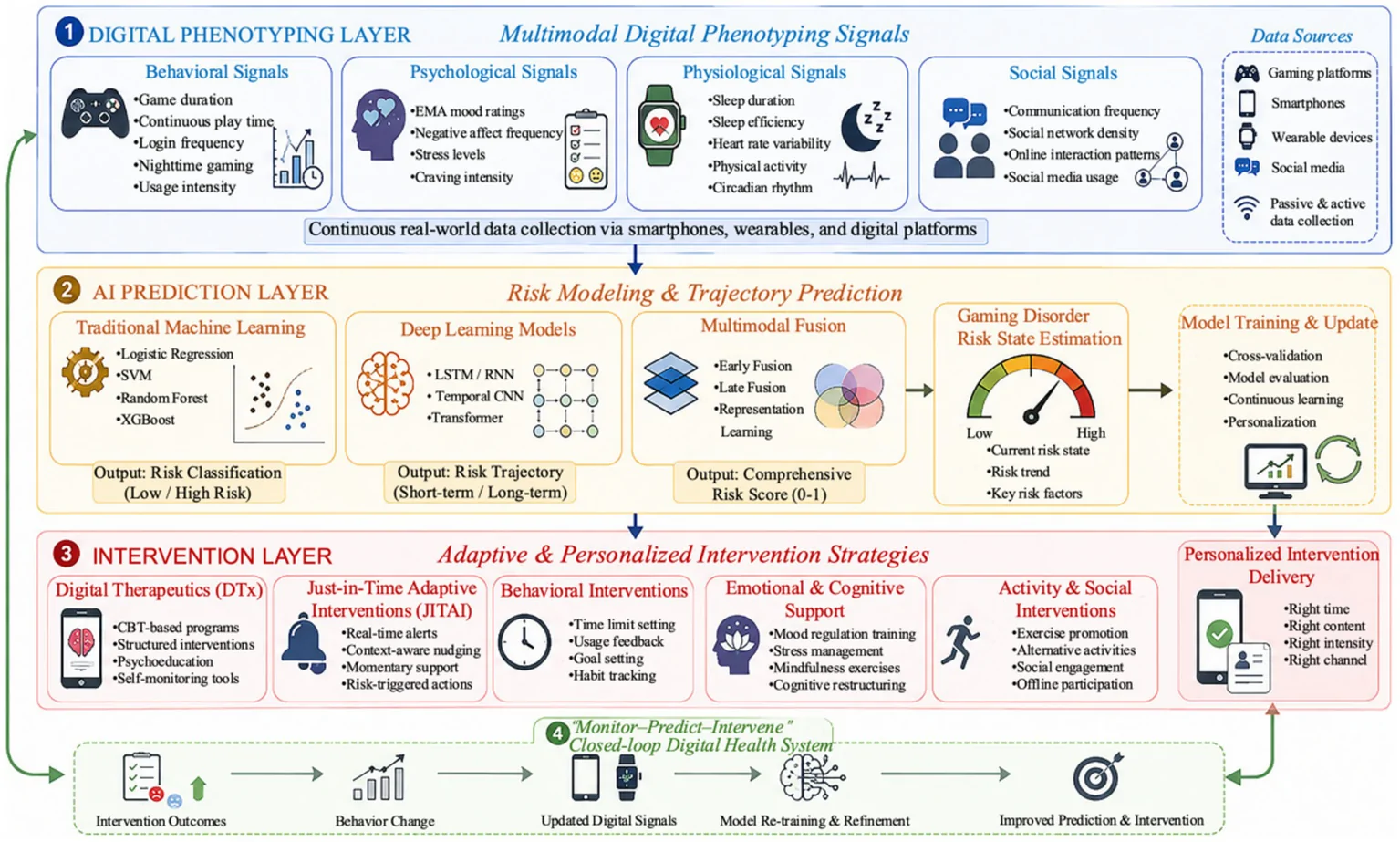
Closed-loop digital health framework for gaming disorder risk monitoring, prediction, and intervention. This framework integrates multimodal digital phenotyping, machine learning-based risk prediction, and adaptive interventions into a continuous closed-loop system. Real-world behavioral, psychological, physiological, and social data are collected, analyzed, and used to deliver personalized interventions, whose outcomes are fed back to continuously optimize prediction models and intervention strategies.

Despite the rapid growth of related research in recent years, existing reviews mainly focus on the epidemiological characteristics, risk factors, neurobiological mechanisms, and traditional intervention strategies of gaming disorder. There is a lack of systematic integration of the translation chain between digital phenotypic surveillance, AI risk prediction, and precision intervention. Furthermore, these emerging technologies are driving the transformation of gaming disorder management from symptom-based passive identification to data-driven proactive prevention, gradually forming a closed-loop digital health model centered on “Monitor–Predict–Intervene.” Therefore, this review systematically summarizes digital phenotypic analysis in gaming disorder, evaluates AI-driven risk prediction methods, and integrates emerging precision intervention strategies. By integrating these components into a “Monitor–Predict–Intervene” framework, this review provides a conceptual basis for continuous risk-oriented management of gaming disorder, supporting early identification, precision prevention, and personalized management while informing future digital mental health research and clinical practice (Figure 1).

## 2 Digital phenotype-driven gaming disorder risk monitoring

### 2.1 From static diagnosis to dynamic risk monitoring

For a long time, the identification of gaming disorder has mainly relied on traditional methods such as questionnaires, self-rating scales, and clinical interviews (7). While traditional assessment methods have important value in screening and diagnosis, these approaches are essentially static and intermittent assessments, reflecting only the symptom state at a specific point in time and failing to depict the dynamic changes in risk. In contrast, the development of gaming disorder does not occur suddenly, but rather evolves gradually along a continuous spectrum from normal use to problematic behavior and then to functional impairment (13). This process is continuously influenced by multiple factors such as emotional state, stress level, sleep rhythm and social environment (14). Assessments at a single point in time are insufficient to meet the needs of early identification and risk warning. Therefore, digital mental health is gradually shifting from disease diagnosis to risk monitoring (15). Continuous, objective, and dynamic tracking of behavior and mental state in the real world provides the possibility for early identification of potentially high-risk individuals.

### 2.2 Core risk dimensions revealed by digital phenotyping

Digital phenotypes enable the quantification of gaming disorder risk by continuously collecting real-world behavioral data from multiple digital devices (16). Studies have shown that the core risk signals can be summarized into four dimensions. The behavioral dimension includes behavioral characteristics such as increased gaming time, increased nighttime gaming, and disrupted circadian rhythms, which directly reflect changes in gaming intensity and control (17). The physiological dimension is based on indicators such as sleep duration, sleep quality, activity level, and heart rate variability (HRV) from wearable devices, which reflect an individual’s physiological recovery state and circadian rhythm stability (18). The psychological dimension captures dynamic changes such as mood fluctuations, stress levels, and gaming cravings through ecological ephemeral assessment (EMA), which helps reduce recall bias (19). The social dimension includes the frequency of social interactions, social network structure, and differences in online and offline behavior, which reflect changes in social function and social dependence patterns (20). The above multidimensional data together constitute the digital representation basis of gaming disorder risk.

### 2.3 Multimodal integration and risk trajectory modeling

Single-dimensional digital features often fail to fully depict complex risk states. Multimodal fusion can integrate behavioral, physiological, psychological, and social information to construct a more complete individual risk profile. For example, an increase in gaming time alone is insufficient to indicate increased risk (21). However, when it occurs simultaneously with reduced sleep, increased negative emotions, and decreased HRV, it is more likely to indicate risk accumulation (4). The research paradigm has further expanded from risk identification to risk trajectory modeling (22). It focuses on the evolution of individual risk over time, including different paths such as risk increase, stabilization, or decrease (23). This dynamic perspective helps identify key turning points, thus providing a basis for precise intervention. Therefore, digital phenotypes are gradually shifting from single-feature descriptions to multi-source information integration and time series modeling, realizing a transformation from risk identification to risk prediction.

## 3 Artificial intelligence-driven risk prediction for gaming disorder

### 3.1 From correlation analysis to risk prediction

Traditional research on gaming disorder primarily relies on cross-sectional surveys and correlation analyses. These studies identify risk factors by examining the relationship between gaming duration, login frequency, nighttime gaming behavior, and gaming motivation with gaming disorder symptoms (24). While this type of research helps understand the mechanisms underlying gaming disorder, its core objective is to explain the phenomenon rather than predict risk. In recent years, the development of digital phenotypes and AI has shifted gaming disorder research from interpretive modeling to predictive modeling (25). Digital phenotypes continuously record an individual’s behavioral changes in the real world, rather than a single point in time, allowing researchers to capture early warning signs before symptoms appear, such as increased nighttime gaming, decreased sleep, and reduced social interaction. Based on this continuous behavioral data, AI models can not only identify the current risk status but also predict future risk trends, providing support for early identification and precise prevention.

### 3.2 Risk classification driven by traditional machine learning

Machine learning is a widely used framework in gaming disorder risk prediction research (26). Traditional supervised learning models identify high-risk individuals by learning relationships between digital phenotyping features and disease status (27). Common methods include logistic regression (LR), support vector machine (SVM), random forest (RF), and extreme gradient boosting (XGBoost). In practice, researchers first extract behavioral features from digital phenotyping data (9). Features include average gaming duration, consecutive gaming days, nighttime gaming proportion, app usage frequency, sleep variability, and social interaction frequency (11). These features are then used in supervised learning models to classify high-risk and low-risk users. In smartphone-based studies, random forest models integrating gaming patterns, sleep regularity, and device usage outperform univariate analyses in risk identification (25). Similarly, XGBoost shows high predictive accuracy due to its nonlinear modeling and feature selection capabilities (28). Compared with traditional statistical methods, these models capture complex variable interactions and improve risk identification. However, traditional machine learning approaches rely on manual feature engineering, requiring predefined behavioral indicators. Although this improves interpretability, it is limited in handling high-dimensional, continuous, and dynamic data and cannot fully capture temporal behavioral evolution.

### 3.3 Deep learning and risk trajectory prediction

With the increasing scale and complexity of digital phenotyping data, deep learning models have become important tools for gaming disorder risk prediction (29). Compared with traditional machine learning, deep learning can learn feature representations directly from raw data and is well suited for time-series and high-dimensional multimodal data (30). Recurrent neural networks (RNNs) and long short-term memory networks (LSTM) are widely used for time-series prediction (31). Gaming behavior shows strong temporal dependencies. LSTM can capture long-term dependencies in behavioral evolution and risk evolution patterns such as increasing nighttime gaming, reduced sleep, and daytime impairment, and predict future gaming disorder risk (32). Temporal convolutional networks (TCNs) have also been applied in behavioral health prediction (33). Compared with RNNs, TCNs offer higher computational efficiency and better capture long-range temporal patterns, making them suitable for large-scale behavioral data (34). Transformer models based on attention mechanisms are transforming time-series modeling (35). Transformers capture long-range dependencies across extended time spans, making them suitable for long-term behavioral trajectories (36). Transformers can link academic stress, emotional fluctuations, and gaming escalation for improved risk prediction. Therefore, deep learning models not only identify current risk but also predict future risk trajectories, including stable, increasing, or worsening patterns. This shift from state classification to trajectory prediction represents a key direction in digital mental health.

### 3.4 Multimodal fusion and integrated risk scoring

Gaming disorder is a complex behavioral addiction shaped by interacting behavioral, psychological, physiological, and social factors (2). A single data source is often insufficient to characterize true risk states. Therefore, multimodal fusion has become an important direction for improving predictive performance. Multimodal risk prediction models integrate four categories of digital phenotyping signals: behavioral, psychological, physiological, and social. Behavioral features include gaming duration, login frequency, and usage patterns (37). Psychological features include EMA-based emotions, stress, and craving (22). Physiological features include sleep duration, HRV, and activity levels (38). Social features include online interactions, network structure, and social media use (39). Common fusion strategies include early fusion, late fusion, and representation learning approaches. Early fusion integrates features before modeling. Late fusion combines outputs from separate unimodal models. Representation learning approaches learn latent cross-modal representations to capture complementary information. Studies show that multimodal models integrating behavioral, sleep, and emotional data outperform unimodal models (40). Gaming behavior alone cannot distinguish heavy recreational gamers from at-risk individuals (41). Adding sleep disturbance and negative affect improves discrimination. In practice, multimodal models often output a continuous risk score (0–1) (42). Higher scores indicate higher risk of future gaming disorder onset. For example, a score of 0.20 indicates low risk, whereas 0.80 indicates high risk. Compared with binary outputs, continuous scores better reflect risk severity and support personalized intervention decisions.

### 3.5 Risk state assessment and individual risk profiling

The goal of risk prediction models is not only predictive accuracy but also actionable information for clinical decision-making (43). Therefore, risk state estimation systems based on model outputs have become an important direction in digital health research. Risk state assessment typically includes three dimensions: current risk, risk trajectory, and key contributing factors. First, systems assess the current risk state of individuals. Individuals can be classified into low-, medium-, and high-risk groups based on integrated scores (44). Second, systems estimate risk trajectories, including increasing, decreasing, or stable patterns. Compared with single time-point assessment, trajectory analysis enables earlier detection of risk changes. For example, a moderately risky but worsening profile may require more urgent intervention than a high-risk but improving profile (16). Third, systems identify key drivers of risk change (45). Risk escalation may be driven by increased nighttime gaming and reduced sleep, or by negative affect and social isolation. Individualized risk profiles help explain risk mechanisms and support targeted interventions. Therefore, risk state assessment bridges digital phenotyping and digital intervention and is central to precision mental health management.

### 3.6 Explainable AI and continual learning framework

Although AI models demonstrate strong performance in risk prediction, the broader clinical application remains limited by the “black box” problem (46). To improve model transparency and credibility, explainable artificial intelligence (XAI) methods are increasingly being introduced into digital mental health. It helps to understand the model’s decision-making process by identifying key features that influence prediction outcomes. Common interpretability methods include shapley additive explanations (SHAP), local interpretable model-agnostic explanations (LIME), and attention-based visualization techniques (47). These methods identify the most influential features driving model predictions. Meanwhile, digital health environments are inherently dynamic, and individual behavioral patterns continuously evolve due to aging, environmental changes, and platform updates (48). Therefore, future models should incorporate continual learning capabilities, enabling ongoing optimization with newly generated data rather than relying on static training datasets (49). Therefore, future predictive systems should also possess continuous learning capabilities, enabling them to constantly update model parameters and maintain predictive performance using newly generated data. Further enhancements will be made to improve model generalization capabilities through cross-group, cross-platform, and cross-cultural validation. This is expected to build an intelligent predictive framework that combines interpretability, adaptability, and clinical usability, and drive gaming disorder management further from risk identification to precise intervention.

## 4 Precision intervention: a closed-loop digital health management system

### 4.1 From risk prediction to real-time intervention

With the development of digital phenotyping and AI, gaming disorder research is shifting from risk identification toward precision intervention (50). Digital mental health has proposed closed-loop frameworks such as Monitor–Predict–Intervene (51). Closed-loop digital health systems achieve a shift from disease treatment to risk prevention through the integration of continuous monitoring, risk prediction, and dynamic intervention (52). In this framework, digital phenotypes are responsible for real-time data collection, artificial intelligence models perform risk assessment, and intervention modules dynamically adjust strategies based on changes in risk, thereby providing timely support before symptoms worsen.

### 4.2 The digital transformation of gaming disorder interventions

Digital therapeutics (DTx) is an important development direction in the field of digital health, providing standardized, repeatable, and sustainable behavioral interventions through evidence-based digital programs (53). In gaming disorder, DTx is mainly based on cognitive behavioral therapy (CBT) (54), covering core modules such as cognitive restructuring, self-monitoring, impulse control, behavior substitution, and time management (55). DTx also integrates psychoeducational content, including knowledge about gaming disorder, sleep hygiene, stress management, and guidance on healthy lifestyles, to improve risk awareness and self-management abilities (56). DTx has been successfully applied in several mental and behavioral health conditions. reSET is approved for substance use disorder (57), Somryst for chronic insomnia (58), and EndeavorRx is the first FDA-approved digital therapeutic for attention-deficit/hyperactivity disorder (ADHD) (59). This demonstrates the feasibility of implementing long-term behavioral interventions on digital platforms and provides an important reference for the clinical translation of gaming disorder.

### 4.3 Multidimensional digital intervention strategies

Gaming disorder is influenced by a combination of behavioral, emotional, physiological, and social factors. Single intervention methods often fail to meet complex needs. Therefore, digital health integrates multidimensional intervention strategies encompassing behavior, emotions, lifestyle, and social support (60). Behavioral intervention aims to reduce excessive gaming behavior and promote healthy gaming habits through self-monitoring, goal setting, time management, and feedback mechanisms (61). The system can generate weekly reports, provide usage feedback, and set phased reduction targets based on risk levels (62). Emotional and cognitive interventions primarily target stress, negative emotions, and difficulties in emotion regulation, improving psychological adaptability through mindfulness training, emotion management, and short-term interventions based on cognitive behavioral therapy (63). Lifestyle interventions reduce the negative impacts of sedentary behavior and irregular schedules by increasing physical activity, improving sleep quality, and participating in offline activities (64). The platform promotes healthy behaviors through exercise incentives, sleep management, and alternative activity recommendations (65). Social support interventions promote social connection through family involvement, peer support, and real-world social interaction, reducing the risk of social isolation and enhancing the maintenance effect of long-term behavioral changes (66).

### 4.4 Just-in-time adaptive intervention and AI-driven personalized intervention optimization

Despite the abundance of intervention options offered by digital therapies and multidimensional interventions, significant differences exist among individuals in terms of risk profiles, behavioral patterns, and intervention needs. Therefore, Just-in-Time Adaptive Intervention (JITAI) is gradually becoming an important direction in the development of digital mental health. JITAI continuously integrates digital phenotypic information and AI risk assessment results to provide individuals with the most suitable intervention support at the appropriate time, rather than relying on a fixed schedul (67). AI provides technical support for JITAI (68). First, AI can identify the optimal intervention time (69), triggering intervention promptly when risk signals such as increased nighttime gaming, decreased sleep, or worsening mood appear. Second, AI can match the most suitable intervention content based on an individual’s risk profile (70). For example, stress-driven individuals are more suited to emotion management strategies, while habit-driven individuals require more behavioral control and time management training. Furthermore, the system can dynamically adjust the intervention intensity according to the risk level and implement interventions through various means such as mobile notifications, chatbots, or digital platforms (71). Overall, JITAI has constructed a closed-loop management model that integrates continuous monitoring, risk prediction, and dynamic intervention, serving as an important pathway to realizing the “Monitor-Predict-Intervene” framework. Although its application in the field of gaming disorder is still under development, it is widely recognized as an important direction for future precision digital mental health management.

## 5 Discussion

This review systematically summarizes digital phenotypes, artificial intelligence prediction models, and digital intervention strategies in gaming disorder, and proposes a comprehensive framework based on a “Monitor–Predict–Intervene” closed-loop digital health system. The field is undergoing a transition from traditional static assessments to a dynamic, data-driven paradigm, moving from symptom identification based on questionnaires and clinical interviews toward risk monitoring and predictive intervention grounded in continuous behavioral data. This transition not only changes data acquisition approaches but, more importantly, drives a structural shift in gaming disorder research. The field is shifting from an outcome-oriented diagnostic model to a process-oriented dynamic modeling paradigm. However, this paradigm remains at an early stage of integration, with heterogeneous levels of maturity and clinical validation across technological modules.

Within this emerging paradigm, digital phenotyping enables continuous quantification of real-world behavior, allowing dynamic capture of multidimensional signals, including gaming behavior, sleep patterns, emotional fluctuations, and social interactions, thereby providing foundational data for characterizing the temporal evolution of gaming disorder. However, a key unresolved issue is that the mapping between these behavioral signals and underlying psychopathological constructs lacks stable theoretical and empirical grounding. Specifically, whether digital behavioral indicators retain consistent clinical interpretability across different contexts remains uncertain. This “behavior–construct mapping instability” represents a fundamental psychometric challenge in digital mental health and limits the interpretability and generalizability of digital phenotyping across application settings.

AI methods further drive a shift from traditional statistical association analysis toward risk prediction and trajectory modeling. This enables the integration of high-dimensional, multimodal behavioral data for individualized risk assessment and dynamic forecasting. However, most current models are trained on specific datasets. These models still have limited external generalization ability across platforms, populations, and real-world settings, reflecting a significant problem with varying data distributions. In addition, although multimodal fusion methods are theoretically expected to enhance information complementarity, the performance gains are often unstable in practice. Multimodal fusion methods are susceptible to data heterogeneity, temporal asynchrony, and sampling bias, thereby introducing additional uncertainty. Therefore, the field remains in a transitional stage, shifting from validation of predictive performance toward validation of generalization robustness.

DTx and JITAI provide key technological pathways for building a “Monitor–Predict–Intervene” closed-loop system. This enables predictive outputs to be translated into dynamic intervention strategies, thereby promoting a shift in gaming disorder management from linear workflows to feedback-driven systems. However, a stable causal mapping between predictive models and intervention strategies remains lacking. Importantly, improvements in predictive performance do not necessarily translate into behavioral change or improved clinical outcomes. The disconnect between prediction and intervention is a key limitation of current closed-loop systems. Furthermore, the application of JITAI in gaming disorder remains at the proof-of-concept or early pilot stage, with its long-term effectiveness and real-world scalability yet to be systematically validated in large-scale randomized controlled trials.

Nevertheless, closed-loop digital health systems demonstrate key structural advantages, including the integration of monitoring, prediction, and intervention through continuous feedback mechanisms, thereby enabling real-time responsiveness and adaptive regulation of dynamic behavioral changes. However, in practical applications, these systems still face multidimensional challenges, including insufficient alignment between digital phenotyping data and clinical constructs, limited cross-domain generalization, and inadequate algorithmic explainability. In addition, continuous behavioral data collection raises concerns regarding privacy and data governance, which have become critical issues across digital mental health populations (72). Moreover, difficulties in integrating these systems into clinical workflows, along with declining adherence to long-term interventions, further limit the real-world feasibility.

Future research should move from single-method optimization toward systematic framework reconstruction, with a particular emphasis on time-trajectory–based dynamic modeling approaches to more precisely characterize the onset and progression of gaming disorder. Meanwhile, multimodal foundation models and federated learning approaches are expected to improve the stability and generalizability of cross-platform data modeling. Furthermore, recent advances in blockchain technology have enabled new paradigms for secure, decentralized, and privacy-preserving data management (73, 74). The integration of blockchain with artificial intelligence, machine learning, deep learning, and federated learning provides potential solutions for trustworthy model training, data governance, and cross-platform collaboration, which may facilitate the development of scalable digital health systems for gaming disorder management (75). At the methodological level, the introduction of causal inference frameworks and digital twin models may help overcome the current correlation-centric analytical paradigm, enabling mechanistic explanations of relationships between behavioral changes and psychological states. At the intervention level, future work should systematically validate the long-term effectiveness and clinical translational potential of digital intervention strategies through real-world randomized controlled trials, thereby enabling a paradigm shift from prediction-driven to mechanism-driven approaches.

## 6 Conclusion

In conclusion, this study focuses on integrating digital phenotyping, AI-driven risk prediction, and adaptive digital intervention into a closed-loop “Monitor-Prediction-Intervention” digital health system for the treatment of gaming disorder. By connecting real-time monitoring, dynamic risk assessment, personalized intervention, and iterative feedback, this framework provides a conceptual foundation for continuous risk-oriented management. It supports the shift from static assessment to longitudinal monitoring, from passive treatment to proactive prevention, and from standardized intervention to individualized precision care. Although numerous methodological and translational challenges remain, the “Monitor-Prediction-Intervention” closed-loop digital health system provides a new theoretical basis for integrating emerging digital technologies into the management of gaming disorder.
